# Prevalence of paediatric inflammatory bowel disease in Sweden: a nationwide population-based register study

**DOI:** 10.1186/s12876-017-0578-9

**Published:** 2017-01-31

**Authors:** J. F. Ludvigsson, K. Büsch, O. Olén, J. Askling, K. E. Smedby, A. Ekbom, E. Lindberg, M. Neovius

**Affiliations:** 1 Department of Paediatrics, Örebro University Hospital, Örebro, Sweden; 2 Department of Medical Epidemiology and Biostatistics, Karolinska Institutet, SE-171 76 Stockholm, Sweden; 3 Department of Medicine Solna, Clinical Epidemiology Unit, Karolinska Institutet, Stockholm, Sweden; 4 Department of paediatric gastroenterology and nutrition, Sachs’ Children and Youth Hospital, Stockholm, Sweden; 5 Department of Rheumatology, Karolinska University Hospital, Stockholm, Sweden

**Keywords:** Prevalence, Paediatrics, Inflammatory bowel disease, Crohn’s disease, Ulcerative colitis, Sweden

## Abstract

**Background:**

We evaluated the impact of different case definition algorithms on the prevalence of paediatric inflammatory bowel disease (IBD), Crohn’s disease (CD) and ulcerative colitis (UC) and to compare the occurrence of certain diseases compared to matched controls.

**Methods:**

Paediatric patients (<18 years) were identified via ICD codes for UC and CD in Swedish registers between 1993 and 2010 (*n* = 1432). Prevalence was defined as ≥2 IBD-related visits. Prevalence of treated children in 2010 was defined as ≥2 IBD-related visits with one visit and ≥1 dispensed IBD-related drug prescription in 2010. To test the robustness of the estimates, prevalence was also calculated according to alternative case definitions. The presence of rheumatic, hepatobiliary, pancreatic, and dermatologic diseases were compared with age-/sex-/county-of-residence-matched general population controls.

**Results:**

The IBD prevalence was 75/100,000 (CD: 29/100,000; UC: 30/100,000; patients with IBD-U: 16/100,000). Prevalence of treated disease in 2010 was 62/100,000 (CD: 23/100,000; UC: 25/100,000; patients with IBD-U: 13/100,000). When age restrictions were employed, the prevalence estimate decreased (<17y: 61/100,000, <16y: 49/100,000 and <15y: 38/100,000).

Compared to general population controls (*n* = 8583), children with IBD had a higher prevalence of dermatologic (4.7% vs. 0.6%), hepatobiliary (including primary sclerosing cholangitis) (5.5% vs. 0.1%), pancreatic (1.7% vs. 0%) and rheumatic diseases (7.2% vs. 1.2%; all *P* < 0.01).

**Conclusions:**

The overall prevalence of paediatric IBD in Sweden was similar to that in earlier regional cohorts. IBD patients had a higher prevalence of comorbid conditions than matched general population controls.

**Electronic supplementary material:**

The online version of this article (doi:10.1186/s12876-017-0578-9) contains supplementary material, which is available to authorized users.

## Background

Inflammatory bowel disease (IBD) is a group of chronic inflammatory disorders, mainly affecting the gastrointestinal tract with extraintestinal manifestations and associated immune disorders. It is usually divided into the phenotypes Crohn’s disease (CD), ulcerative colitis (UC) and IBD unclassified (IBD-U, a form of colonic IBD whose features make it impossible to define as either CD or UC at diagnosis). IBD is associated with substantial morbidity and decreased quality of life in patients [1]. The frequently intensive disease course results in significant use of healthcare resources including outpatient visits, hospitalization and surgery [2].

Although most patients are adults, some 10–25% are diagnosed before 21 years of age [1, 3–5]. Prevalence estimates for paediatric IBD range from 16 to 58 per 100,000 (see Additional file 1: Table S1 for estimates) [6–11]. However, the reported figures are difficult to compare due to differences in data collection and analysis [12].

The prevalence of a disease is important for planning of health care and allocation of clinical resources. The prevalence of IBD in Sweden across all age groups has been reported to be 650 per 100,000 with 1/3 seeing a physician and receiving treatment in a given year [13]. However, it is important to report prevalence of paediatric IBD separately, since these patients are taken care of by paediatric gastroenterologists and not adult gastroenterologists, since early onset IBD (IBD diagnosis <10 years of age [14]) may have a more aggressive or complicated clinical course [15] and since paediatric IBD seems to have increased over time [4, 6]. In addition, data on extra-intestinal manifestations and other immune-mediated conditions in IBD will increase knowledge about the disease burden in these patients [16, 17].

The primary aim of this study was to describe the prevalence of paediatric IBD, CD and UC in Sweden by evaluating the impact of different register-based definitions of IBD on prevalence estimates. The secondary aim was to examine the prevalence of certain comorbidities in paediatric IBD (i.e., extra-intestinal manifestations) vs. matched general population controls.

## Methods

### Setting

In 2010, Sweden had a population of 1.9 million <18 years of age (Statistics Sweden [18]). The Swedish healthcare system is tax funded and offers universal access.

### Identification of children with inflammatory bowel disease

The Swedish National Patient Register was used to identify all patients <18 years of age in 2010 with a visit listing a diagnosis of CD (ICD10 K50; ICD9 555) or UC (ICD10 K51; ICD9 556; see supplemental digital content (SDC), Additional file 2: Table S2). This register was launched in 1964, became nationwide in 1987, and includes non-primary outpatient physician visits since 2001. Visits to general practitioners (i.e., primary care in Sweden) are not included [19]. Register linkage was performed using the unique personal identity number [20].

Prevalent cases of IBD were defined as children living in Sweden in 2010 with ≥2 visits in either inpatient care (1993–2010) or non-primary outpatient care (2001–2010, including day surgery since 1997). Patients with listings of both CD and UC diagnoses were included in the overall IBD prevalence estimate and defined as IBD-U. To estimate the number of children with treated disease in 2010, we identified children with ≥2 IBD-related visits, of which ≥1 occurred in 2010 plus ≥1 dispensed drug prescription for aminosalicylates, corticosteroids, or immunosuppressants in 2010. To test the robustness of the estimates, prevalence was also calculated according to alternative case definitions based on the total number of visits with a listing of CD or UC, time interval between visits, type of diagnosis (main or contributory), IBD-related treatment. To be able to compare results with previous reports, prevalence was also estimated using age restrictions (<15 years, <16 years and <17 years) [7–10].

### Identification of matched general population controls

Up to six general population controls were matched by age, sex, and county of residence to each child at the time of first IBD diagnosis (defined as 1st registered CD or UC diagnosis in inpatient or non-primary outpatient care). The matched general population controls were sampled from the Register of the Total Population [18].

This register covers the entire Swedish population and includes information on age, sex, and place of residence as well as dates of birth, death, and emigration status.

### Medical and surgical treatment

To quantify IBD-related healthcare resource use during the study period as well as current use in 2010, data regarding surgical procedures and medical treatment were collected from the National Patient Register using surgical procedure codes see and the Prescribed Drug Register using ATC codes.

Major surgery was defined as total colectomy, partial excision of intestine, and partial excision of rectum and minor intestinal surgery as dilatation of the intestine, stricturoplasty, sphincterotomy, suture and procedure related to fistulae, fissures or abscesses (see SDC, Additional file 3: Table S3). Medical treatment data included dispensed prescriptions of aminosalicylates, corticosteroids, and immunosuppressants (see SDC, Additional file 4: Table S4). Other IBD-related treatment such as exclusive enteral nutrition (EEN) is not captured in the Swedish national registers and could therefore not be evaluated. Overall, >99% of non-infusion drug use (including biologics) is captured in the Prescribed Drug Register, but infusion biologics are covered to a lesser extent (e.g., some 20% of infliximab use in 2009) [21]. We also examined dispensed prescriptions 2005–2010 as well as during 2010 only.

### Presence of other diseases

To compare the prevalence of certain other conditions in IBD patients to matched controls, information on those diseases considered to be extra-intestinal manifestations [22] was obtained through the National Patient Register during the study period (for a complete list see SDC, Additional file 5: Table S5), and included rheumatic diseases, dermatologic diseases, hepatobiliary diseases including primary sclerosing cholangitis, and pancreatic diseases [22]. Presence of any of those conditions during the study period (1993–2010) was obtained using inpatient and non-primary outpatient care using disease-specific ICD codes (≥1 visit) [17].

### Statistics

Prevalence of IBD, CD, UC, and IBD-U was defined as the prevalence on December 31st 2010, and calculated as the number of children (<18 years) alive and residing in Sweden on December 31st 2010 with at least two diagnosis listings of CD or UC during the study period (1993–2010) divided by the total Swedish population <18 years of age on that date (*n* = 1,919,094) [18]. Prevalence estimates of physician-diagnosed and coded comorbidities in children with IBD (including CD, UC, and IBD-U) and those diseases in matched controls were compared using the chi-square test.

All statistical analyses were performed using SAS (version 9.4, SAS Institute Inc., Cary, NC, USA).

### Ethics

Ethical approval for this study was granted by the Regional Ethics Committee, Stockholm, Sweden [23]. Permission to use the databases in this study was granted by the two government agencies the *National Board of Health and Welfare* and *Statistics Sweden*.

## Results

In the National Patient Register, 1432 children (see SDC, Additional file 6: Figure S1; mean age in 2010: 14 years; standard deviation: 3; range 2–17 years; 56% boys) were identified as having a history of at least 2 visits listing an IBD diagnosis during the study period. Of those, 1209 children had at least 1 IBD-related visit in 2010 and 1193 children had at least 1 IBD-related visit in 2010 and dispensed ≥1 IBD-related drug prescriptions in 2010 (treated disease in a given year; see SDC, Additional file 7: Figure S2).

Patient characteristics for the 1432 children are described in Table 1 and Additional file 8: Figure S3.

**Table 1:** Characteristics, medical and surgical treatment of children with register-identified inflammatory bowel disease (prevalent cases Dec 31st, 2010)

	Inflammatory Bowel Disease *n* = 1432	Crohn’s Disease *n* = 548	Ulcerative Colitis *n* = 585	IBD-U *n* = 299
Boys, n (%)	802 (56%)	319 (58%)	317 (54%)	166 (56%)
Age (y); mean (SD)				
*at identification*	10 (4)	11 (4)	10 (4)	9 (4)
*in 2010*	14 (3)	14 (3)	14 (3)	14 (3)
Age groups in 2010				
*0–9 years* ^d^	156 (11%)	51 (9%)	71 (12%)	34 (11%)
*10–17 years*	1276 (89%)	497 (91%)	514 (88%)	265 (89%)
Presence of diseases considered to be extra-intestinal manifestations	281 (20%)	115 (21%)	91 (16%)	75 (25%)
IBD-related in- and outpatient visits in 2010				
*Outpatient visits*	1199 (84%)	451 (82%)	492 (84%)	256 (86%)
*Inpatient visits*	310 (22%)	128 (23%)	121 (21%)	61 (20%)
Major IBD-related surgery				
In 2010	35 (2%)	23 (4%)	7 (1%)	5 (2%)
During 1993-2010^c^	78 (5%)	28 (5%)	23 (4%)	27 (9%)
Colectomy	36 (3%)	1 (0.2%)	18 (3%)	17 (6%)
Intestinal surgery	45 (3%)	27 (5%)	5 (1%)	13 (4%)
Rectal surgery	6 (0.4%)	1 (0.2%)	2 (0.3%)	3 (1%)
Minor bowel surgery	83 (6%)	58 (11%)	6 (1%)	19 (6%)
IBD-related drug treatment				
In 2010	1213 (85%)	443 (80%)	502 (86%)	262 (88%)
*Aminosalicylates*	1 065 (74%)	370 (68%)	472 (81%)	223 (75%)
*Steroids*	593 (41%)	203 (37%)	257 (44%)	133 (44%)
*Immunosuppressants (incl. biologics)* ^a^	666 (47%)	307 (56%)	209 (36%)	150 (50%)
During 2005-2010^b^	1333 (93%)	500 (91%)	541 (92%)	292 (98%)

IBD = inflammatory bowel disease; Patients required to have at least two listings of the selected diagnoses to be defined as a case; Differential diagnosis defined as Behcet’s disease, irritable bowel syndrome, intestinal tuberculosis, amoebic colitis, celiac disease, diverticulitis, ischemic colitis, non-infective colitis, radiation damage, and infectious/bacterial colitis. Diseases with main focus on those considered to be extra-intestinal manifestations were defined as rheumatic, dermatologic, hepatobiliary and pancreatic diseases and were based on the World Gastroenterology Organization Practice Guidelines for the diagnosis and management of IBD and/or the treatment guidelines for IBD from the Swedish Association of Gastroenterology [22, 29]. Major IBD-related surgery defined as total colectomy, partial excision of intestine, and partial excision of rectum; IBD-related drug treatment defined as dispensed prescription of aminosalicylates, corticosteroids, immune modifiers, and biologics; Codes used for physician-diagnosed and comorbid conditions considered to be extra-intestinal manifestations are available in the SDC, Additional file 5: Table S5; Codes used in this analysis are available above in Additional file 3: Table S3 (surgical procedures) and Additional file 4: Table S4 (dispensed prescription drugs); Numbers do not sum up as overlap between the groups e.g., colectomy and intestinal surgery or steroids and immunosuppressants were possible

^a^Infusion biologics are covered to a lesser extent in the Prescribed Drug Register (e.g., about 20% of infliximab use in 2009) [21]; Given that infusion biologics are covered to a lesser extent, this number should be interpreted with caution

^b^Data from the Prescribed Drug Register only available since 2005 onwards

^c^Note that surgery after the 18th birthday is not counted and that only a minority of patients in the sample have been followed for >4 years at their 18th birthday

^d^Early onset defined as with a diagnosis before 10 years of age [14]

Of all children with IBD, 20% had at least one recorded comorbidity considered to represent extra-intestinal manifestations.

### Prevalence

**Fig. 1:**
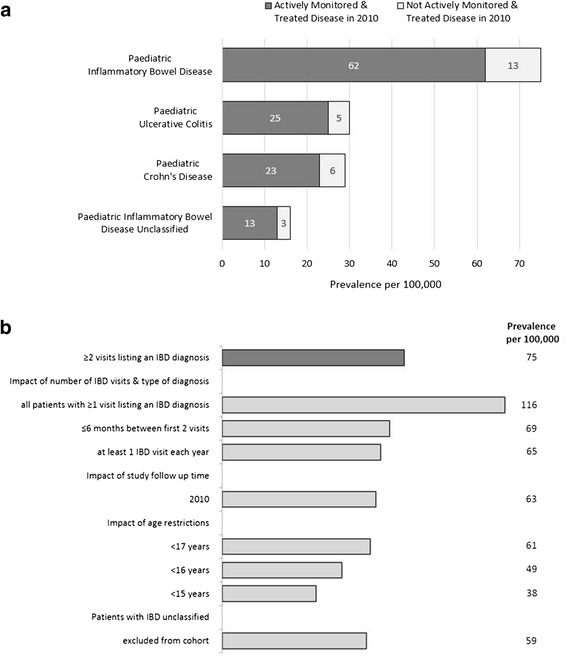
**a** Register-based prevalence of paediatric inflammatory bowel disease, ulcerative colitis and Crohn’s disease (<18 years of age, *n* = 1432) for actively monitored and treated disease in 2010 and overall in 2010. IBD = inflammatory bowel disease; UC = ulcerative colitis; CD = Crohn’s disease; Patients required to have at least 2 listings of the selected diagnoses to be defined as a case; Treated in a given year was defined as having at least one of the two inflammatory bowel disease-related visits occurring in 2010 and at least one inflammatory bowel disease-related dispensed prescription in 2010. **b** Additional sensitivity analysis to evaluate the impact on the overall inflammatory bowel disease prevalence estimate. Estimate based on a source population in 2010 of 1,919,094 < 18 years old, 1,794,267 < 17 years old, 1,675,031 < 16 years old and 1,564,959 < 15 years old (www.scb.se); IBD = inflammatory bowel disease

The prevalence of paediatric IBD in 2010 was 75 per 100,000 (30 per 100,000 for UC, 29 per 100,000 CD, 16 per 100,000 for patients with IBD-U on December 31st 2010; Fig. 1a). When restricting the case definition to children treated in 2010, the prevalence estimate was 62 per 100,000 (Fig. 1a). Disease specific prevalence estimates for treated UC, CD and for patients with IBD-U during the study period were 25 per 100,000, 23 per 100,000 and 13 per 100,000.

### Sensitivity analysis (Fig. 1b; Additional file 7: Figure S2)

Requiring only 1 visit, the overall prevalence estimate of IBD increased from 75 per 100,000 to 116 per 100,000.

Shortening the time period of data capture to 2010 reduced the prevalence to 63 per 100,000 (−16% compared to the base case). Adding the requirement of a maximum of 6 or 12 months between the first visits, the prevalence estimates were 69 per 100,000 and 72 per 100,000 for 6 and 12 months (−8 and −4% compared to the base case), respectively. Requiring at least 1 IBD-related visit every year during follow up lead to a prevalence of 65 per 100,000 (−14% compared to the base case prevalence estimate).

Restricting the analysis to *<17* years, as in a previous US study [7], we estimated an IBD prevalence of 61 per 100,000. When restricting the analysis to ages *<16* years as in two previous Swedish studies [8, 9], the prevalence decreased from 75 per 100,000 to 49 per 100,000. And finally restricting the analysis to *<15* year-old children as in a previous Danish study [10] we found a prevalence of 38 per 100,000.

### Inflammatory bowel disease-related healthcare use

In 2010, 84% of 1432 the children had one or more IBD-related visits in non-primary outpatient care and 22% had at least one inpatient care admission (see Table 1).

Five percent of the 1432 children with IBD had undergone at least one major IBD-related surgical procedure during the study period (1993–2010). In patients with a major surgical procedure the mean and median time from first recorded IBD diagnosis to first major IBD-related surgery were 1.5 and 1.4 years, respectively (quartile range 0.5–2.4 years). In the total IBD cohort, the mean and median observation times from register-identification to December 31st, 2010, were 4 and 3 years, respectively. Intestinal surgery was the most common surgical procedure performed in CD patients (5%), colectomy the most common one in UC patients (3%). In 2010, 2% of the 1432 children had at least one major surgical procedure.

Medical drug treatment penetration was high with 85% of children having had at least one dispensed prescription for aminosalicylates, corticosteroids, or immunosuppressants in 2010 and 93% during 2005–2010 (see Table 1).

### Presence of other diseases e.g., those considered to be extra-intestinal manifestations

**Fig. 2:**
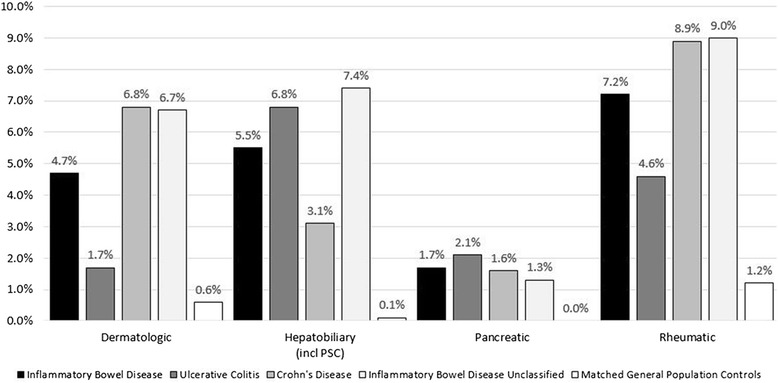
Presence of diseases considered to be extra-intestinal manifestations in prevalent children with inflammatory bowel disease (*n* = 1432), ulcerative colitis (*n* = 585), Crohn’s disease (*n* = 548), IBD unclassified (*n* = 299) and in matched general population controls (*n* = 8583; matched by age, sex, and county of residence at time of identification). * *p* < 0.01 for comparison between children with IBD, UC; CD or IBD-U and matched controls; Patients required to have at least 2 listings of the selected diagnoses to be defined as a case; Rheumatic diseases defined as rheumatoid arthritis, psoriatic and enteropathic arthropathies, juvenile arthritis, unspecified arthritis, pain in joints, Sjögrens syndrome, Behcet’s disease, ankylosing spondylitis; Dermatologic diseases defined as pyoderma gangraenosum, erythema nodosum, psoriasis, febrile neutrophilic dermatosis, aphthous stomatitis; Hepatobiliary diseases defined as primary sclerosing cholangitis, pericholangitis, cholelithiasis, chronic active hepatitis, nonalcoholic fatty liver disease; Pancreatic diseases defined as acute or chronic pancreatitis; Codes used in this analysis are available in the SDC, Additional file 5: Table S5; patients IBD-U defined as patients with both a UC and a CD diagnosis during follow up

IBD patients had a higher prevalence of physician-reported dermatologic, hepatobiliary, pancreatic and rheumatic disease than matched general population controls (all *P* < 0.01; Fig. 2).

Of the conditions investigated in the 1432 IBD children, rheumatic diseases were most common (7.2%), followed by hepatobiliary diseases (5.5%) and dermatologic diseases (4.7%). A higher percentage of children with CD than UC had dermatologic (6.8% versus 1.7%; *P* < 0.01) or rheumatic diseases (8.9% vs. 4.6%; *P* < 0.01), while the opposite was the case for hepatobiliary diseases (3.1% versus 6.8%; *P* < 0.01).

## Discussion

The overall prevalence of paediatric IBD in 2010 was 75 per 100,000, while the prevalence of treated IBD in 2010 was estimated to be 62 per 100,000 overall, 23 per 100,000 for CD, 25 per 100,000 for UC, and 13 per 100,000 for patients with IBD-U). Extra-intestinal manifestations were, as expected, considerably more common in IBD than in the general population, with rheumatic disease being the most common comorbidity in CD (8.9%) and hepatobiliary conditions in UC (6.8%).

### Previous research

There are few prevalence studies on paediatric IBD so far (Additional file 1: Table S1) [6–10]. A recent Canadian study of 3169 patients found a similar prevalence of paediatric IBD (58.3 per 100,000) compared to 75 per 100,000 in our study [6]. In contrast a US study [7] provided lower prevalence estimates for UC (19.5 per 100,000) and CD (12.0 per 100,000) (Additional file 1: Table S1).

As shown in several of our sensitivity analyses, the between-study variations likely arise from differences in study methods, including case ascertainment, IBD definition, length of data capture periods, age distributions, and age cut-off points rather than true prevalence variations. Even though our base case estimate was quite robust varying only between 62 and 75 per 100,000 when testing the clinically most relevant algorithms and definitions, the estimates increased to 116 per 100,000 when only 1 visit was required for case definition and decreased to 38 per 100,000 when the analysis was restricted to children <15 years. The increase in prevalence from 38 to 75 per 100,000 < 18 years can be explained by factors such as the longer data capture period for older children, which increases the chance of registration of a diagnosis. Disease onset is usually more common in older than younger children [24, 25] thus inclusion of older adolescents will increase prevalence estimates [6]. By analysing the paediatric subgroup in more detail, we found that only about 1/3 of *all* IBD patients are treated in a given year (i.e., having had ≥2 IBD-related visits with one visit and ≥1 dispensed IBD-related drug prescription in 2010) [13], compared to 83% in children. Also, as previously shown, the percentage of patients with a IBD-U was 21% in children compared to 17% overall [13].

A recent cross-sectional study using outpatient and inpatient insurance claims data found that IBD patients <20 years of age have more immune-mediated diseases than matched controls [17]. The authors of that study found a positive association between CD and rheumatoid arthritis (odds ratio [OR] 15.7, 95% CI 4.6–53.7), systemic lupus erythematosus (OR 41.0, 95% CI 2.3–719.1) and hypothyroidism (OR 2.9, 95% CI 1.4–6.1). UC was associated with a higher prevalence of diabetes (OR 2.7, 95% CI 1.1–6.6). Both in our study and in that of Kappelman et al. [17] rheumatic diseases were the most common extra-intestinal manifestations. In the prospective, observational study from Dotson et al., 17% of the included 1009 children <16 years with newly diagnosed IBD displayed extra-intestinal manifestations [16]. This percentage increased to 28% during follow-up (26 months) [16]. We found that about 20% of IBD patients <18 years of age had ≥1 physician-diagnosed rheumatic, hepatobiliary, pancreatic or dermatologic disease. However, due to differences in definitions and study design, results between studies are difficult to compare. While information regarding extra-intestinal manifestations was prospectively collected in regular scheduled visits in the study by Dotson et al. [16], we used register-based information to obtain the prevalence of certain conditions in IBD patients and their matched controls. Using registry-based information may have underestimated their true prevalence as some gastroenterologists regard those diseases as being part of IBD (i.e., extra-intestinal manifestations) and therefore do not assign a specific ICD code for, e.g., rheumatic disease.

### Strengths

A strength of this study was the access to routinely collected nationwide data on inpatient and non-primary outpatient care, as well as dispensed prescription drugs. The large sample size allowed us to assess variations in prevalence and to perform sensitivity analyses. Using a register-based approach we were able to assess the overall prevalence of any paediatric IBD and IBD requiring active monitoring and treatment. The latter estimate may be of more practical interest as it better reflects the actual burden to the healthcare system in a given year. The prevalence helps to describe the overall burden of a chronic, relapsing and remitting disease such as IBD as it includes children in remission who might be in need of resources and care at a later point in time. Additional strengths included that there was no need to adjust the prevalence to allow generalizability or to extrapolate to the general population as Swedish registers are national and virtually complete [19].

### Limitations

A limitation of this register-based approach pertains to the specificity and the sensitivity of our IBD definition since our diagnoses were not validated through clinical examinations. Although we did not have access to symptom and endoscopy data to confirm the IBD diagnoses, our earlier review on the National Patient Registry found a positive predictive value (PPV) of 85–95% for most diagnoses [19]. Data from patient chart reviews suggest a PPV of 93% for ≥2 recorded diagnoses with IBD in Sweden [26]. This is almost identical to the PPV of 92% found when British researchers evaluated the General Practice Research Database in 2002 [27], another source of registry-based research.

Another limitation is that data from non-primary outpatient care were not available for the whole study period. However, we believe that the sensitivity of IBD in the National Patient Register was high for children as paediatric patients are managed by hospital-based specialists rather than general practitioners and are closely monitored with specialist visits every 3 to 6 months (92% of the identified children with IBD were seen every 3 to 6 months in 2010) and 1238 patients (86%) had ≥1 IBD-related visit every year during follow up. Testing the impact of a more liberal definition defined as only ≥1 visit for the case definition increased the prevalence estimate to 116 per 100,000 in 2010. To minimize the risk of false positive cases, we therefore decided to use ≥2 visits in our main analysis even though this meant that we probably excluded some true cases. An additional analysis showed that of the 662 patients with only 1 visit until 2009, 7% had an additional visit in 2010 while 93% were not seen by a physician for their IBD. Therefore, our approach most likely underestimates the prevalence even though we consider the impact to be small. A stricter definition such as the one used in the Canadian study [6] was considered to be too strict as the Swedish National Patient Register only includes inpatient and non-primary outpatient specialist visits, and not contacts with primary healthcare providers.

We identified a high percentage of patients with both diagnostic codes for CD and UC registered, likely including patients with IBD-U, indeterminate colitis, patients who had a disease onset typical of UC and who later developed clear signs of CD or patients who even got the wrong IBD diagnosis at some point because of a typo. Given our lack of endoscopy or histology data, and the fact that many patients have to be classified as IBD-U, even with all clinical information available [28], we analysed children with both diagnoses listings during the study period as a separate group since we could not say with certainty which diagnoses they really had. In our previous analysis of all age-groups we analysed the impact of re-classification of patients with both diagnoses of UC and CD during the study period using the last 9 diagnoses and showed that impact on the prevalence would be small [13]. However, when trying to describe those patients in more detail, e.g., regarding occurrence of extra-intestinal manifestations, we excluded them due to the resulting diagnostic ambiguity. Our higher estimate compared to previous studies is probably partly explained by a longer data capture period increasing the chance of getting both diagnoses (our study had a capture period of 17 years compared to 2–14 years in previous studies [6–8, 10]).

We had near complete coverage of dispensed non-infusion prescriptions (including e.g., azathioprine and adalimumab), but unfortunately were only able to identify some 20% of infusion biologics (e.g., infliximab), which makes it important to point out that the recorded proportion of patients on infusion biologics in our dataset is probably lower than in reality. Moreover, we were not at all able to identify treatment with EEN in the registers.

## Conclusions

In conclusion, we found a prevalence of treated paediatric IBD in 2010 of 62 per 100,000 and a total prevalence of 75 per 100,000. The prevalence was stable once ≥2 or more IBD-related visits were required, except for age-restricted estimates. While 93% of children were treated with aminosalicylates, corticosteroids, or immunosuppressants, 5% had a history of major IBD-related surgery. As previously observed, dermatologic, hepatobiliary, pancreatic and rheumatic disease were more common in IBD than in the general population.

## Additional files

Additional file 1: Table S1. Summary of paediatric prevalence studies – by year of publication. (PDF 107 kb)
Additional file 2: Table S2. Summary of ICD codes used for ulcerative colitis and Crohn’s disease. (PDF 36 kb)
Additional file 3: Table S3. Summary of surgical procedure codes used in the analysis. (PDF 86 kb)
Additional file 4: Table S4. Summary of codes used for medical treatment/prescribed drugs. (PDF 47 kb)
Additional file 5: Table S5. Summary of codes used for extra-intestinal manifestations/comorbidities. (PDF 151 kb)
Additional file 6: Figure S1. Paediatric inflammatory bowel disease patient flowchart. (JPG 323 kb)
Additional file 7: Figure S2. Additional sensitivity analysis to evaluate the impact on the overall inflammatory bowel disease prevalence estimate. (JPG 345 kb)
Additional file 8: Figure S3. Age at identification and age in 2010 for prevalent cohort (Dec 31st 2010; *n* = 1432). (JPG 153 kb)

## Abbreviations

ATC: Anatomical therapeutical chemical
CD: Crohn’s disease
IBD: Inflammatory bowel disease
ICD: International classification of disease
PPV: Positive predictive value
SDC: Supplemental digital content
UC: Ulcerative colitis
