# Supplementary material for: Prevalence of paediatric inflammatory bowel disease in Sweden: a nationwide population-based register study
Source: BMC Gastroenterol. 2017 Jan 31;17:23. doi: 10.1186/s12876-017-0578-9 (PMC5282815; doi:10.1186/s12876-017-0578-9)
Supplement: Additional file 1: Table S1. — Summary of paediatric prevalence studies – by year of publication. (PDF 107 kb) [file 12876_2017_578_MOESM1_ESM.pdf]

Table S1 Su mmary of paediatric prevalence studies* – by year of publication

Author	Year	Country / region	n	Age group	Year of data collection	IBD prevalence per 100 000	CD prevalence per 100 000	UC prevalence per 100 000
**Paediatric prevalence studies**								
Hildebrand et al[1]	1991	Sweden	333	<16y	1984-1985	17.6	6.2	7.5
Hildebrand et al[2]	1994	Sweden, Southwestern	132	<16y	1983-1987	21.5		
Jakobsen et al[3]	2008	Denmark, Eastern part	98; 145	<15y	1998-2000; 2002-2004	15.8; 20.3	6.7; 8.2	8.3; 10.5
Benchimol et al[4]	2009	Canada, Ontario	3169	6m-18y	1994–2005	58.3	32.8–36.2±	20.3–22.1±
Abramson et al[5]	2010	USA, Northern California	675	<17y	1996-2006		12.0	19.5
El-Matary et al[6]	2014	Canada, one province		<17y	1978-2007	33.9	18.9	12.7
Current study	2016	Sweden	1432; 1193	<18y	1993-2010	Cumulative: 75 Treated in 2010: 62	Cumulative: 29 Treated in 2010: 23	Cumulative: 30 Treated in 2010: 25
**Overall prevalence studies reporting paediatric prevalence estimates separately**								
Kappelman et al[7]	2013	USA, four census regions		<20y	2008-2009		58	34
Loftus et al[8]	2007	USA, Olmsted County, Minnesota	81	<20y	1940-2000		114.9	106.7
Bernstein et al[9]	2006	Canada, 5 provinces		<20y	1998-2000		30.5 (MB) - 71.1 (AB)	17.5 (BC) - 30.7 (AB)
Current study	2016	Sweden		<20y	1993-2010	105	40	43

IBD = inflammatory bowel disease; CD = Crohn's disease; UC = ulcerative colitis; AB = Alberta; MB = Manitoba; BC = British Columbia

* Publications presenting only overall prevalence estimates for entire populations (both adult and paediatric) or with focus on adults only were excluded; Comparison of results is hampered by differences in data collection techniques, case ascertainment (e.g. ICD10, diagnostic criteria of Lennard-Jones, histologic classification described by Morson and Dawson and Yardley and Donowitz), IBD definition (e.g. IBD defined as UC+CD+probable CD+IC (Hildebrand et al 1991); IBD defined as UC+CD+IC excl. infectious or other specific underlying enteritis or colitis (Jakobsen et al 2008), IBD defined as UC+CD plus parts of unclassifiable patients)), and measurements; In the paper by Bernstein et al (2006) the province abbreviations represent: British Columbia (BC), Alberta (AB), and Manitoba (MB).

± The lower number in the range represents prevalent cases excluding unclassifiable patients. The upper range of prevalence represents the prevalence including one-third of unclassifiable patients (except in the 6 months–4 years age group, where two-thirds of unclassifiable patients are included as possible cases).

References to eTable 1

1. Hildebrand H, Fredrikzon B, Holmquist L, Kristiansson B, Lindquist B: **Chronic inflammatory bowel disease in children and adolescents in Sweden**. *J Pediatr Gastroenterol Nutr* 1991, **13**(3):293-297.
2. Hildebrand H, Brydolf M, Holmquist L, Krantz I, Kristiansson B: **Incidence and prevalence of inflammatory bowel disease in children in south-western Sweden**. *Acta Paediatr* 1994, **83**(6):640-645.
3. Jakobsen C, Wewer V, Urne F, Andersen J, Faerk J, Kramer I, Stagegaard B, Pilgaard B, Weile B, Paerregaard A: **Incidence of ulcerative colitis and Crohn's disease in Danish children: Still rising or levelling out?** *Journal of Crohn's & colitis* 2008, **2**(2):152-157.
4. Benchimol EI, Guttmann A, Griffiths AM, Rabeneck L, Mack DR, Brill H, Howard J, Guan J, To T: **Increasing incidence of paediatric inflammatory bowel disease in Ontario, Canada: evidence from health administrative data**. *Gut* 2009, **58**(11):1490-1497.
5. Abramson O, Durant M, Mow W, Finley A, Kodali P, Wong A, Tavares V, McCroskey E, Liu L, Lewis JD *et al*: **Incidence, prevalence, and time trends of pediatric inflammatory bowel disease in Northern California, 1996 to 2006**. *J Pediatr* 2010, **157**(2):233-239 e231.
6. El-Matary W, Moroz SP, Bernstein CN: **Inflammatory bowel disease in children of Manitoba: 30 years' experience of a tertiary center**. *J Pediatr Gastroenterol Nutr* 2014, **59**(6):763-766.
7. Kappelman MD, Moore KR, Allen JK, Cook SF: **Recent trends in the prevalence of Crohn's disease and ulcerative colitis in a commercially insured US population**. *Dig Dis Sci* 2013, **58**(2):519-525.
8. Loftus EV, Jr.: **The burden of inflammatory bowel disease in the United States: a moving target?** *Clin Gastroenterol Hepatol* 2007, **5**(12):1383-1384.
9. Bernstein CN, Wajda A, Svenson LW, MacKenzie A, Koehoorn M, Jackson M, Fedorak R, Israel D, Blanchard JF: **The epidemiology of inflammatory bowel disease in Canada: a population-based study**. *Am J Gastroenterol* 2006, **101**(7):1559-1568.
