# Supplementary material for: Prevalence of paediatric inflammatory bowel disease in Sweden: a nationwide population-based register study
Source: BMC Gastroenterol. 2017 Jan 31;17:23. doi: 10.1186/s12876-017-0578-9 (PMC5282815; doi:10.1186/s12876-017-0578-9)
Supplement: Additional file 2: Table S2. — Summary of ICD codes used for ulcerative colitis and Crohn’s disease. (PDF 36 kb) [file 12876_2017_578_MOESM2_ESM.pdf]

eTable 2 Summary of ICD codes used for ulcerative colitis and Crohn's disease

	**ICD9 (1987-1996)**	**ICD10 (since 1997)**
**Crohn's disease**	555	K50
**Ulcerative colitis**	556	K51
