# Supplementary material for: Prevalence of paediatric inflammatory bowel disease in Sweden: a nationwide population-based register study
Source: BMC Gastroenterol. 2017 Jan 31;17:23. doi: 10.1186/s12876-017-0578-9 (PMC5282815; doi:10.1186/s12876-017-0578-9)
Supplement: Additional file 3: Table S3. — Summary of surgical procedure codes used in the analysis. (PDF 86 kb) [file 12876_2017_578_MOESM3_ESM.pdf]

eTable 3 Summary of surgical procedure codes used in the analysis

	National classification of procedures (till 1996)	NOMESCO classification of surgical procedures (since 1997)
**Total colectomy / Ileostomy**	4650- 4654,<br>4700	JFH
**Partial excision of intestine**	4630, 4631, 4640-4649	JFB
**Partial excision of rectum**	4820- 4828	JGB
**Minor surgical procedures e.g. stricturoplasty**	4960, 4740, 4741, 4910, 4611, 4010, 4900, 4922, 4970, 4971	JFA60, JFA38, JFA58, JGA58, JHD00, JFA70, JFA71, JFA80, JFA81, JHD10, JFA76, JFA86, JHA00, JHA20, JHD20, JHD30, JHD33
