# Supplementary material for: Prevalence of paediatric inflammatory bowel disease in Sweden: a nationwide population-based register study
Source: BMC Gastroenterol. 2017 Jan 31;17:23. doi: 10.1186/s12876-017-0578-9 (PMC5282815; doi:10.1186/s12876-017-0578-9)
Supplement: Additional file 4: Table S4. — Summary of codes used for medical treatment/prescribed drugs. (PDF 47 kb) [file 12876_2017_578_MOESM4_ESM.pdf]

eTable 4 Summary of codes used for medical treatment / prescribed drugs

	ATC codes
**Aminosalicylic acid and similar agents**	A07EC
**Corticosteroids**	A07EA, H02BX, H02AB
**Immunomodulators (incl. biologics), purine analogues**	L04A, L01BB02
