# Supplementary material for: Prevalence of paediatric inflammatory bowel disease in Sweden: a nationwide population-based register study
Source: BMC Gastroenterol. 2017 Jan 31;17:23. doi: 10.1186/s12876-017-0578-9 (PMC5282815; doi:10.1186/s12876-017-0578-9)
Supplement: Additional file 5: Table S5. — Summary of codes used for extra-intestinal manifestations/comorbidities. (PDF 151 kb) [file 12876_2017_578_MOESM5_ESM.pdf]

eTable 5 Summary of codes used for extra-intestinal manifestations / comorbidities

	ICD9 (1987-1996)	ICD10 (since 1997)
**Rheumatic / Metabolic**<br>Incl. rheumatoid arthritis, psoriatic and enteropathic arthropathies, juvenile arthritis, unspecified arthritis, pain in joints, Sjögren's syndrome, Behcet's disease, and ankylosing spondylitis	268C, 710C, 711C, 713, 714A, 714B, 714C, 714D, 714W, 719D, 720A, 730A, 730C, 733A	M05, M06.0, M06.2, M06.3, M06.8, M06.9, M07, M08, M09, M12.3, M13.9, M25.5, M35.0, M35.2, M45, M80, M83.8, M86.0, M86.9
**Dermatologic**<br>Incl. pyoderma gangraenosum, erythema nodosum, psoriasis, febrile neutrophilic dermatosis, and aphthous stomatitis	528A, 528B, 528C, 686A, 695C, 696	K12.0, K12.1, L40, L52, L88, L98.2
**Hepatobiliary**<br>Incl. primary sclerosing cholangitis, pericholangitis, cholelithiasis, chronic active hepatitis, and nonalcoholic fatty liver disease	571E, 571W, 573C, 573D, 574, 576B	K73.2, K75.8, K75.9, K76.0, K80, K83.0
**Pancreatic**<br>Incl. acute pancreatitis or other chronic pancreatitis	577A, 577B	K85, K86.1
