# Supplementary figures and images for: Prevalence of paediatric inflammatory bowel disease in Sweden: a nationwide population-based register study
Source: BMC Gastroenterol. 2017 Jan 31;17:23. doi: 10.1186/s12876-017-0578-9 (PMC5282815; doi:10.1186/s12876-017-0578-9)

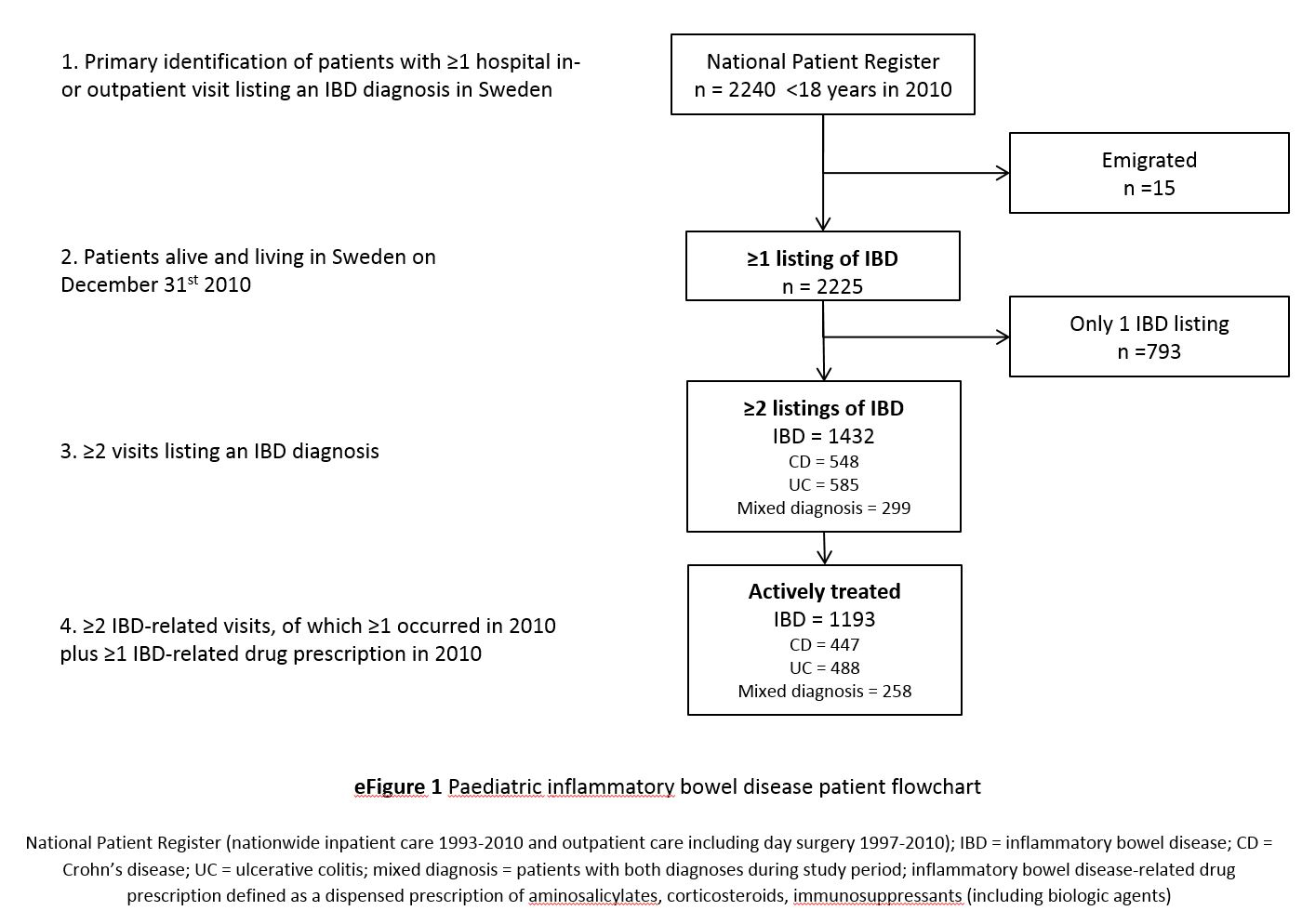

Supplement: Additional file 6: Figure S1. — Paediatric inflammatory bowel disease patient flowchart. (JPG 323 kb) [file 12876_2017_578_MOESM6_ESM.jpg]

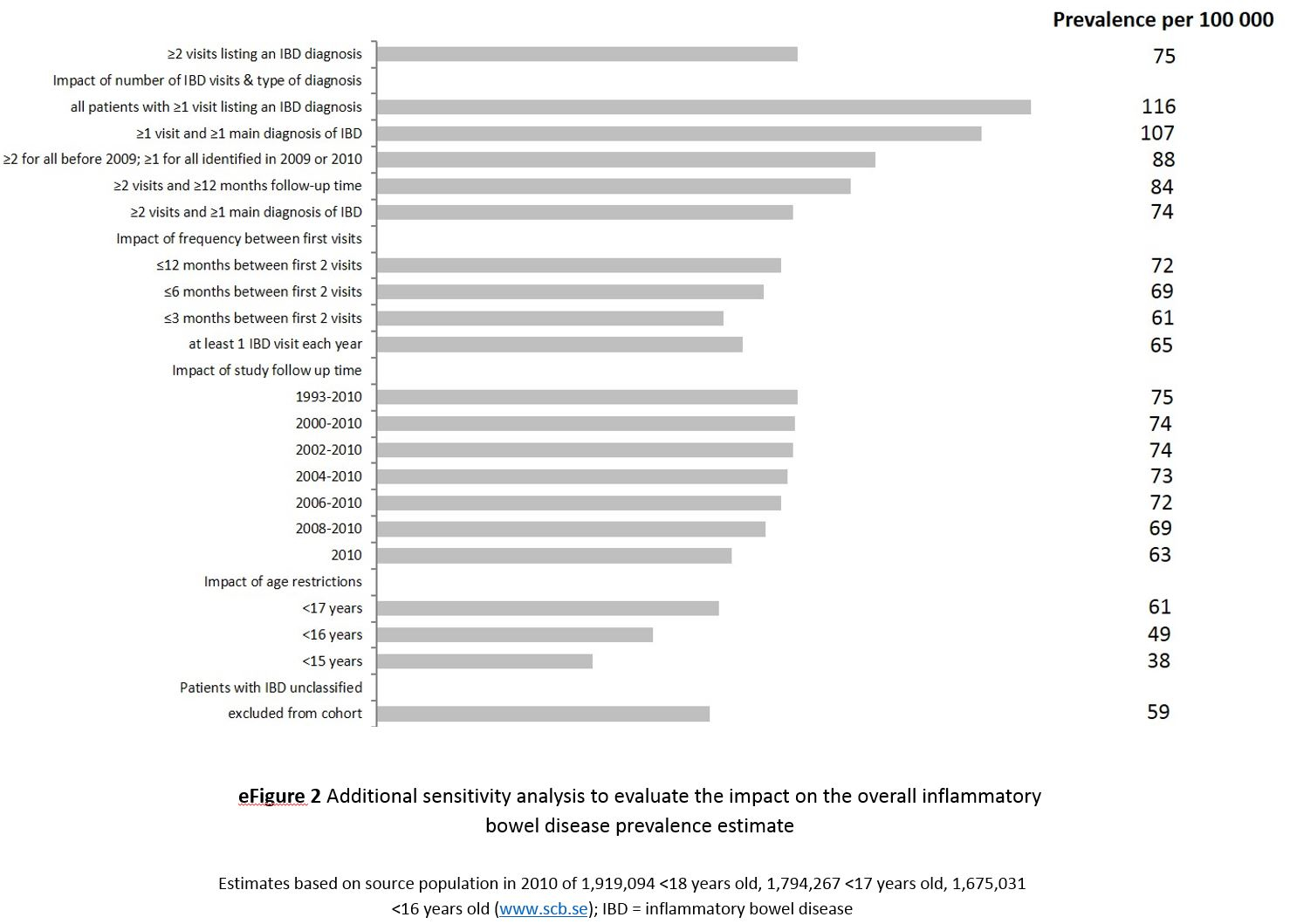

Supplement: Additional file 7: Figure S2. — Additional sensitivity analysis to evaluate the impact on the overall inflammatory bowel disease prevalence estimate. (JPG 345 kb) [file 12876_2017_578_MOESM7_ESM.jpg]

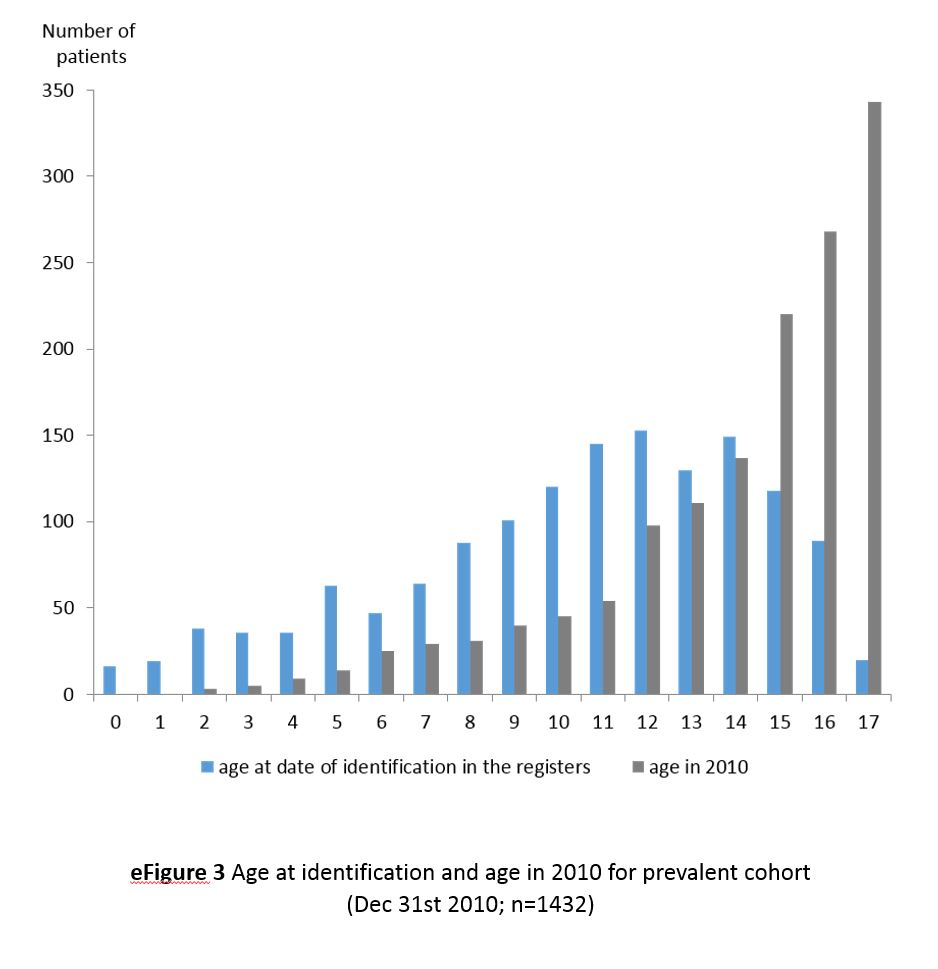

Supplement: Additional file 8: Figure S3. — Age at identification and age in 2010 for prevalent cohort (Dec 31st 2010; n = 1432). (JPG 153 kb) [file 12876_2017_578_MOESM8_ESM.jpg]
